# Menstrual hygiene management and fertility in Togo: Exploring the causal pathways

**DOI:** 10.1177/17455057261458614

**Published:** 2026-06-04

**Authors:** Anusree Paul, Salmata Ouedraogo, Anastasie Amboulé Abath

**Affiliations:** 1 Department of Economics and Administration, Université du Québec à Chicoutimi, Chicoutimi, QC, Canada; 2 Department of Education, Université du Québec à Chicoutimi, Chicoutimi, QC, Canada

**Keywords:** menstrual hygiene management, fertility rate, control function approach, endogeneity, Togo, I12, I19, C3

## Abstract

**Background:**

Menstrual hygiene management (MHM) remains a neglected concern in Togo, despite its critical importance for the education, health, and dignity of girls and women. Neglecting MHM directly impacts women’s reproductive morbidities that may adversely affect fertility outcomes. However, there is limited literature on MHM practices in Togo and their impact on individual fertility.

**Objectives:**

The study aims to examine the (1) effects of structural determinants—namely WASH infrastructure, socio-economic disparities, and social norms on menstrual hygiene practices, and (2) causal effect of MHM on reproductive health outcome, measured in terms of fertility of Togolese women. Considering the endogeneity of MHM, we aim to provide evidence that the roles of WASH facilities, social norms, and socio-economic factors influence MHM and thereby linking it directly to fertility of women.

**Design:**

The study uses secondary data from a cross sectional survey of nationally representative Multiple Indicator Cluster Survey (MICS6, 2017) for Togo.

**Methods:**

We have used control function (CF) approach to address the endogeneity of MHM to estimate the causal inference of MHM on the fertility of ever-married women aged between 15-49 years in Togo. The source of endogeneity of MHM is explained through the channels of social exclusion-related unobservable, and availability of WASH facilities.

**Results:**

The access to WASH facilities improve the probability of MHM by 31-53 percentage points. Our study establishes the existence of a strong causal relationship between MHM and fertility rate, establishing the endogeneity of MHM. The fertility increases (4-5%) significantly for women who manage their menstrual hygiene.

**Conclusion:**

Effective MHM is crucial for individual dignity, health, and well-being. Our study indicates that proper menstrual hygeine management has significantly affect the reproductive health of women measured in terms of fertility. To foster an *enabling environment of MHM concerned*, the study recommended a few public policy interventions.

## Plain language summary

Menstrual hygiene management remains a neglected concern in Togo. Neglecting menstrual hygiene directly impacts women’s reproductive health and is closely linked to gender equality and empowerment. However, there is limited literature on menstrual hygiene practices and their impact on fertility rates. Our study aims to fill this gap by providing evidence of the existence of a causal impact of self-reported menstrual hygiene management on the fertility rate of Togolese women. Using the Multiple Indicator Cluster Survey (MICS6) of Togo for 2017, our findings indicate the significance of access to water, hand-washing facilities, and cleaning supplies in improving menstrual hygiene management. Furthermore, we find that socioeconomic factors such as education level, family affluence, etc, influence women’s ability to manage their menstrual hygiene. Our study underscores the need for integrating menstrual hygiene into reproductive health policies and addressing key socio-economic and structural barriers that hinder proper menstrual hygiene management.

## Introduction

Menstrual hygiene management (MHM) and reproductive health are two major public health concerns worldwide, particularly in the context of developing and less-developed economies. Neglecting MHM has consequences that go beyond one’s health and well-being. Even though it isn’t specifically stated in the objectives, it has social ramifications, especially for gender equality and women’s empowerment. It also aligns with numerous Sustainable Development Goals (SDGs), such as health (Goal 3), gender equality (Goal 5), and water and sanitation (Goal 6).^1,2^

Proper management of menstruation is essential for women and girls’ access to education and economic opportunities, as well as for their good sexual and reproductive health and dignity.^3,4^ Beyond using sanitary products, access to secure and private locations where women and girls may quietly and hygienically manage their periods is essential for good MHM. This includes having access to suitable menstruation products’ supplies, soap, and clean water.^5,6^ However, these necessary resources are either unavailable or too expensive in many places, especially in emerging and low-income nations.^7^ Additionally, financial constraints worsen the issue by making it more difficult for women to access high-quality menstruation.^8,9^

The lack of adequate MHM facilities has far-reaching consequences for women’s reproductive health. Reproductive health was defined in 1994 by the International Conference on Population and Development (ICPD) as a condition of total physical, mental, and social well-being in all areas of the reproductive system.^10,11^ This inclusive definition highlights the importance of good menstrual hygiene management to maintain safe and healthy reproductive practices. However, this objective is undermined by poor MHM, which results in poor pregnancy outcomes and infertility/low fertility, among other consequences related to reproductive health.^12^

Existing empirical studies highlight that poor MHM is associated with a variety of reproductive health hazards,^13^ mostly measured in terms of reproductive tract infections (RTI)/reproductive morbidity, sexually transmitted infections (STIs)/sexually transmitted diseases (STDs) etc.,^14–19^ which in turn may lead to infertility or low fertility.^12,20–23^ The existing studies are primarily cross-national and primary survey-based (cross-section observational, case-control, interventional). For example, Anand et al. (2015)^14^ conducted a cross-sectional observational study in India to study the determinants of MHM and how it affects the self-reported RTI and abnormal vaginal discharge. Similarly, Das et al. (2015)^15^ conducted a hospital-based case-control study on 486 women in Odisha, India, and studied the association between MHM and urogenital infections. In more recent studies, Mudi et al. (2023)^17^ showed that poor MHM increases the prevalence of RTI by conducting a primary survey in Odisha, India. Al Karmi et al. (2024)^19^ also showed that poor MHM enhances the chance of RTIs. They conducted a study on refugee women in Jordon. The literature on the association between MHM and reproductive health is quite thin in the context of Africa. Sumpter and Torondel (2013)^7^ showed an association between MHM and RTIs in a systematic review of 14 articles from 2013, including two from Tanzania, but the association is weak, and the methodologies vary greatly. Using a community-based cross-sectional survey, Ademas et al. (2020)^16^ and Akoth et al. (2024)^18^ showed a similar association between MHM and reproductive morbidity in Ethiopia, Kenya, Uganda, Burkina Faso, Ghana and Niger.

Fertility is one of the major indicators of reproductive health constructed by World Health Organisation (1998).^24^ The channels through which MHM affects fertility remain underexplored in the existing literature. One possible channel to link poor MHM with fertility is through RTIs/STIs. However, the existing studies have not explored this channel. The existing literature has shown a link between Poor MHM and RTIs/STIs, and the association between RTIs and infertility/lower fertility – one of the major outcomes of RTIs/STIs.^20–23^ However, the direct linkage between MHM and fertility has yet to be investigated. In our present study, we try to fill this gap by examining the causal effect of MHM on fertility, taking Togo as a case in point.

In social circumstances, menstruation is highly stigmatized where menstruating women’s behaviours are driven by certain rituals and meanings linked with menstruation within a specific system, which eventually (re)produces religion and culture.^25–27^ Further, social taboos that restrict open talks about menstruation and impede women’s and girls’ abilities to manage their periods hygienically and confidently frequently cause embarrassment and constraints.^28,29^ This lack of communication causes poor MHM procedures a widespread ignorance. For example, a study carried out by BIS Afrique (2017)^30^ in Togo among 788 young and adolescent girls of age group 10 – 24 years. They found that girls and women who are illiterate or have primary education have the lowest level of knowledge regarding menstruation and its related hygiene and believed that menstruation is an unclean body waste. These ideas support the stigma and humiliation associated with menstruation, which makes it challenging for women and girls to discuss and openly handle their periods. In addition to cultural shame and stigmatization, insufficient sanitary facilities in schools and homes exacerbate the difficulties of managing menstruation. Due to a lack of decent bathrooms, washing facilities, and privacy in schools, many girls are forced to stay home during their periods, missing out on significant educational opportunities in countries like sub-Saharan Africa.^31,32^ According to the BIS Afrique (2017)^30^ survey, many girls in Togo were forced to use unhygienic alternatives such as tissues or reusable cloths, which they could not thoroughly clean owing to a shortage of soap and water. Due to this social exclusion (taboos and stigmatizations), women may be prevented from seeking medical attention for menstruation-related health issues, exacerbating health-related dangers. Further, despite menstrual hygiene being formally included under reproductive health, adequate attention was not given to the water, sanitation, and hygiene (WASH), or sexual and reproductive health programs.^33^

The existing empirical studies suffer from some weaknesses, and our study contributes to the existing body of literature in the following ways: First, the causal pathways of MHM and fertility as an indicator of reproductive health have not been studied yet. Proper management of menstruation depends on the water, sanitation, and hygiene (WASH) facilities available,^5,6^ and on the behavior of menstruating women shaped by their religious and cultural beliefs.^25,28^ These factors represent potential pathways for the endogeneity of menstrual hygiene management. However, none of the existing studies have explored how these channels contribute to the endogeneity of MHM and its potential causal impact on reproductive health. The consequence of not addressing the endogeneity issue leads to biased estimations.^34,35^ To the best of our knowledge, our study is the first kind of study in this field that addresses the causal effect of MHM on the fertility,^36^ by accounting for the endogeneity of MHM in case of Togo. We use the Control Function (CF) approach to address this endogeneity.

Second, existing empirical studies mainly used country and location-specific cross-sectional observational/case-control/interventional small-scale data, where generalization of the results is limited. Instead, we have used the Multiple Indicator Cluster Survey (MICS6) database for Togo, which is nationally representative data, and the generalization of the findings for Togo is quite evident.

Given this backdrop, our study aims to examine the following two interconnected objectives: first, we empirically investigate how and to what extent the underlying structural determinants, namely, WASH infrastructure, socio-economic disparities, and social norms affects the menstrual hygiene practices; second, we estimate the causal impact of MHM on reproductive health outcome, measured in terms of fertility of Togolese women. Considering the endogeneity of MHM, we aim to provide evidence that the roles of WASH facilities, social norms, and socio-economic factors influence MHM and thereby link it directly to the fertility of women.

Our findings support that the access to water, hand-washing facilities, and cleaning supplies improve MHM. Socioeconomic factors such as education level, family affluence, and the age of the household head all influence women’s capacity to manage their menstrual hygiene. Importantly, our study finds a strong causal relationship between MHM and fertility rate. After accounting for endogeneity, we find that improving menstrual hygiene management has a favourable and significant influence on fertility, highlighting the significance of treating MHM to promote reproductive health and well-being.

## Methods

### Sample size and power analysis

For this analysis, we rely on cross-sectional household survey of the Multiple Indicator Cluster Survey, round 6 (MICS6) database of Togo for the year 2017 (latest available). The MICS6 survey obtained ethical clearance from the National Ethics Committee under the Togolese Ministry of Health. As this data is available in the public domain, separate ethical approval is not required for this study.

MICS uses two-stage stratified cluster sampling methodology to ensure representation of data at both national and subnational levels. The optimal sample size in MICS follows a precision-based framework, called *power allocation*, which minimizes the margin of error and design effects (DEFF) arising from clustering for the key indicators and population subgroups.^37^ Thus, MICS samples are sufficiently large to generate reliable estimates and ensure valid statistical inference of the estimated relationship. For our study, we have extracted data from the Household Questionnaire, and from the Women’s Questionnaire (aged 15-49 years). The Household Questionnaire provides socioeconomic and environmental covariates, including wealth quintiles, household-level information, WASH infrastructure etc. The Women Questionnaire provides information on fertility, birth history, menstrual hygiene, etc. After merging these two data sets, we find 5% of the data have missing information on MHM. We have treated these missing values using the regression imputation method in STATA 19. We have taken the availability of water facility and wealth are two explanatory variables in the regression method to impute missing values of MHM. After these checks and cleaning, we retained data of 6997 women on which we have imposed our inclusion and exclusion criteria to get our final dataset.

### The exclusion and inclusion criteria

The inclusion criterion involves ever-married women respondents within the age group of 15 – 49 years. The exclusion criterion involves the removal of three categories of respondents: unmarried, respondents who have undergone a hysterectomy, and respondents in their menopausal phase. After removing those observations, our final dataset consists of 4989 data points for ever-married women aged 15—49. The manuscript preparation follows the guidelines of STROBE developed in von Elm et al.(2007).^38^

### Variables

MICS6 survey data provides the number of births per women. Guided by the existing literature, we have taken *children ever born* as our dependent variable, which is the individual level fertility.^39^

The menstrual hygiene management (MHM) variable is constructed as follows: The women questionnaire provides information regarding the menstrual hygiene management of adolescent girls and women of the age group 15- 49 years. The questionnaire primarily focuses on the following three questions:

• *During your last menstrual period were you able to wash and change in privacy while at home? Yes/No*
• *Did you use any materials such as sanitary pads,* tampons, *or cloth? Yes/No*
• *Were the materials reusable? Yes/No*

WHO/UNICEF Joint Monitoring Programme 2012^36^ defined MHM as:

*Women and adolescent girls are using a clean menstrual management material to absorb or collect menstrual blood that can be changed in privacy as often as necessary, using soap and water for washing the body as required, and having access to safe and convenient facilities to dispose of used menstrual management materials. They understand the basic facts linked to the menstrual cycle and how to manage it with dignity and without discomfort or fear.* ^1^

Based on this definition, the above three questions capture the menstrual hygiene management of an individual if the individual replied ‘*Yes*’ to all of them. We have constructed a menstrual hygiene management (MHM) variable, which is a binary and takes the value 1 when the respondent replied ‘*Yes*’ to all three questions asked, 0 otherwise. This is a crude measure of MHM because these questions do not capture the availability of ‘*soap/cleansing materials*’ to clean the body and reusable materials (if they are used); and the access to ‘*safe and convenient facilities to dispose of used menstrual management materials’*. Thus, the MHM variable signifies whether the individual is managing menstrual hygiene or not.

MICS6 survey data also provides information on women’s socioeconomic background, individuals’ exposure to mass media and information and communication technology (ICT), fertility, contraception, etc. The selection of relevant variables in this study was based on their potential impact on MHM and fertility drawing mostly from literature. We have computed and constructed many variables based on the information available in MICS6 database of Togo as explained below:

Apart from controlling the socio-economic factors of the respondent (Age, education level of respondents, residence type, region, ethnicity), we have considered the age, sex, and educational level of the household head to control for social norms, as the imposition of societal norms has a differential impact on men and women-headed households.^40,41^ We further consider the ICT exposure of the respondents, which controls their awareness regarding hygiene and health. The women’s questionnaire also provides information related to cigarette smoking, tobacco, and alcohol consumption, and methods of contraception. We have controlled these factors, as nicotine consumption has an adverse impact on fertility, and the use of contraception positively affects fertility. The constructions of the variables are as follows:

Wealth*:* A quintile of wealth index is provided in the MICS6 data, which is calculated based on a composite measure of a household’s cumulative living standard. The index categorizes households into five quintiles – poorest, poor, average, rich, richest. We have reconstructed this into three categories: rich & richest (coded as 0), average or middle (coded as 1), poor and poorest (coded as 2).

Media/ICT exposure*:* MICS6 data also provides information regarding ICT and media exposure of individuals. Information on frequency of listening radio, watching TV, using of internet, possession of mobile phone, etc are given. It can be argued that ICT and media exposure enhance social awareness related to hygiene and reproductive health.^42^ Media/ICT exposure, binary variable, is constructed as a proxy of social awareness by considering the respondent’s exposure to radio, TV, mobile and internet. We have defined the variable in Table 1.

**Table 1.** Variables descriptions.

Variables	Description
**Outcome Variable**	
Fertility	Total number of children ever born to the respondent
**Endogeneous Variable**	
Menstrual hygiene management	The dummy variable takes the value 1 if the individual is managing menstrual hygiene, 0 otherwise.
**Instrumental Variable**	
WASH facilities available	The categorical variable takes the value 0 with no water, hand washing, and hand cleanser available. It takes the value 1 when water is available at respondent’s own dwelling/yard/plot without a handwashing place and/or hand cleanser. It takes the value 2 when water is available elsewhere without a handwashing place and/or hand cleanser. It takes the value of 3 when water is available elsewhere with a handwashing place and/or hand cleanser. Finally, 4 indicates that water is available at one’s dwelling/yard/plot with hand washing places and/or hand cleansers.
**Other Controls**	
Age of the respondents	Age of the respondent (in years)
Level of education	The categorical value takes 0 if the individual has secondary and above education, 1 for primary education, and 2 for no education.
Wealth status	The categorical variable is based on wealth score. It takes the value 0 for the rich, 1 for average/middle income, and 2 for the poor.
Place/type of residence	The dummy variable takes the value 1 if the individual is residing in a rural area and 0 for urban.
Region of the respondents	We have seven categories of region are as follows: 1= Maritime/Sea, 2= Trays, 3=central, 4= Kara 5= Savannas, 6= Lomé Commune, and 7= Urban Gulf
Ethnicity of the respondents	The religion/ethnicity categories are: 1= Adja-Ewe, 2= Kabye-Tem, 3= Paragourma, 4= Ana-Ife, 5= Akposso/Akébou, 6= Autres togolais, and 7= Autres nationalités
Sex of the household head	The dummy variable takes the value 1 if the head of the household is female, 0 for male
Age of the household head	Age of the household head (in years)
Level of education of the household head	The categorical value takes 0 if the household head has secondary and above education, 1 for primary education, and 2 for no education.
ICT/media exposure of the respondent	The dummy variable takes the value 1 if the individual is exposed to radio listening or TV watching or if she has a mobile phone or has used the internet, and it takes the value 0 if the individual is not exposed to any media/ICT.
Use of contraception method	The dummy takes the value 1 if the individual does not use any method of contraception, and 0 if she uses any contraception method.
Consumption of nicotine (cigarettes/tobacco) or alcohol	The dummy takes the value 1, if the individual has ever tried smoking or non-smokable tobacco products or consumed alcohol, 0 otherwise.

Source: Authors’ compilation based on MICs database of Togo, 2017.

Contraception method: MICS6 women’s questionnaire outlined various contraception methods to avoid pregnancy. We have constructed a binary variable whether the individual has used/adopted any kind of contraception method or not.

Consumption of nicotine and alcohol: The questionnaire provides information regarding the nicotine (cigarette smoking and tobacco/non-smoking) and alcohol consumption of each individual. Literature reveals the adverse impact of these lifestyle habits on fertility.^43^ Hence, we have controlled this factor. We have constructed a dummy variable based on the information on the consumption of these three products, explained in Table 1.

## Endogeneity of MHM

Menstruation and menstrual practices still face social prohibitions, strong adherence to menstrual taboos and traditional beliefs, and parents’ reluctance to openly discuss relevant issues with their daughters have contributed to a lack of correct knowledge of menstrual hygiene practices.^44^ Menstruation among school-age girls and women is a neglected issue in West African nations including Togo. Menstruating girls and women frequently go through their periods in difficult and stigmatizing situations with unsupportive social norms and limitations that prevent them from fully participating in daily activities. Women in Togo were not permitted to cook for men or be in a man’s presence while they were on their periods until recently. In more extreme situations, women were compelled to leave their homes during their periods and wait to return until they were finished.^30^ These social exclusion or unsupportive social norms imposing restrictions on menstruating women are the possible source of the endogeneity of MHM.

### Instrumental variables

WASH Facility: Our construction of variables differs from the existing studies, where the country and geographic location-specific survey questionnaire focuses information on types of handwashing and cleansing of body materials, frequency of bathing, its location, frequency and way of washing during menstruation, etc to capture the factors that affect menstrual hygiene.^6,14–19^ The MICS6 questionnaire covered extensive information on WASH facilities, which includes the availability of water for other uses (other than drinking, and household use), its source, location, hand washing facility, availability of hand cleanser, toilet facility, etc.

Data related availability of water for other uses (other than drinking and for household use), its source, location, hand washing facility, availability of hand cleanser, etc. are provided in the questionnaire. All these variables are highly interrelated with each other. Hence, to avoid possible multicollinearity issues, we have constructed a categorical variable with five categories considering all factors described in Table 1. The existing literature indicates a strong positive association between WASH facilities and MHM.^15,17,18^ We hypothesize that better WASH availability improves menstrual hygiene practices.

### Statistical analysis

We have used a control function (CF) approach to address the endogeneity of MHM to estimate the causal inference of MHM on the fertility of ever-married women aged between 15-49 years in Togo. The possible source of endogeneity of MHM is the omitted variables due to the social exclusion-related unobservable that affect the menstrual hygiene of Togolese women. The CF approach models the dependence between the unobservable on the observables in a way that allows the construction of a control function that is conditional on the observables to address the issue of endogeneity.^35,45,46^ We hypothesize that menstrual health management $\left({MHM}_{i}\right)$ has a significant causal impact on the reproductive health of individuals. We have considered children ever born ( ${CEB}_{i}$ ) as a proxy of the reproductive health status/fertility of individual woman as the outcome variable.

Thus, to define the relationship between fertility ( ${CEB}_{i}$ ) and menstrual health management $\left({MHM}_{i}\right)$ , following Wooldridge (2015), we consider the outcome equation as:

(1) $${CEB}_{i}=X_{1i}\beta +\gamma .{MHM}_{i}+e_{i}$$

Where, ${CEB}_{i}$ is the outcome variable, and ${mhm}_{i}$ is the endogenous binary variable of our interest. $X_{1i}$ is the sub vector of $X_{i}$ , which consists of socioeconomic, social awareness, and other variables of concern of the *i*^*th*^ individual as explained in Table 1. We consider strong exogeneity of $X_{1i}$ , that is $E\left(X_{1i}^{\prime }e_{i}\right)=0.$ β and γ are the vector of parameters to be estimated. $e_{i}$ is the idiosyncratic error term, which is ∼Normal(0,1) .

The selection equation is defined as

(2) $${MHM}_{i}=1\left[X_{i}\alpha +\varepsilon_{i}\geq 0\right]$$

Where, 1[.] is the binary indicator function. $X_{i}$ consists of 2 sets of variables: $W_{i}$ is the WASH facilities (the instruments) and $X_{1i}$ vector of the socio-economic profile, social awareness, and other variables of concern of the *i*^*th*^ individual. α is the vector of parameters to be estimated. $\varepsilon_{i}$ is idiosyncratic error term.

We assume a strong exogeneity of vector $X_{i}$ , that is $E\left(X_{i}^{\prime }\varepsilon_{i}\right)=0,$ and $\left(e_{i},\varepsilon_{i}\right)$ is independent of $X_{i}$ . Further, $e_{i}$ is linearly correlated with $\varepsilon_{i}$ : $E\left(e_{i}|\varepsilon_{i}\right)=\delta .\varepsilon_{i}$ , and ${\;\varepsilon }_{i}∼Normal\left(0,1\right)$ .

As an implication of equations (1), and (2), we consider that ${MHM}_{i}$ follows a probit function as:

(3) $$\mathrm{Pr}\left({MHM}_{i}|X_{i}\right)=\Phi \left(X_{i}\alpha \right)$$

Where Φ(.) is the standard normal cumulative distribution function.

Equation (4) defines the full distribution for ${MHM}_{i}$ , the conditional expectation $E\left({CEB}_{i}|X_{i},{MHM}_{i}\right)$ is defined as:

(4) $$E\left({CEB}_{i}|X_{i},{MHM}_{i},{\hat{m}}_{i}\right)=\exp\left(X_{1i}\beta +\gamma {MHM}_{i}+\delta {\hat{m}}_{i}\right)$$

Where, μ(.)=ϕ(.)/Φ(.) is the inverse Mills ratio, and the term $\hat{m}\left(.\right)$ is called the “generalized residuals” defined as:^47^

(5) $${\hat{m}}_{i}\left({MHM}_{i},X_{i}\alpha \right)={MHM}_{i}\;.\mu \left(\;X_{i}\hat{\alpha }\right)-\left(1-{MHM}_{i}\;\right).\;\mu \left(-X_{i}\hat{\alpha }\right)$$

We use the standard two-step procedure to estimate the model, where in 1^st^ step we run probit of ${mhm}_{i}$ , $X_{i}^{\prime }$ to get $\hat{\alpha }$ to obtain the generalized residual ${\hat{m}}_{i}\left(.\right).$ Substituting ${\hat{m}}_{i}\left(.\right)$ in equation (1), in the second step, we use Poisson to estimate it by using $X_{1i}\;\text{and}\;\hat{m}\left(.\right)$ as instruments.

The Poisson regression framework is applicable in the second-stage estimation, as the dependent variable (CEB) is a non-negative integer count, and therefore, fails to satisfy the distributional conditions of Ordinary Least Squares (OLS) estimation. The Poisson specification effectively ensures positive conditional mean predictions and also accounts for the heteroscedastic nature of the count data. Hence, we have employed Two-Stage Residual Inclusion (2SRI) control function approach by using the generalised residual ( $\hat{m}\left(.\right)$ ) to address the endogeneity of MHM.^48^

Further, to ensure the reliability and validity of our baseline estimates, we have conducted the following robustness and sensitivity tests: first, we have conducted Placebo test^49^ to validate our identification strategy and to confirm the exclusion restriction of the instrumental variables. Under the Placebo test/falsification test, we replace the true outcome variable (fertility) with a ‘placebo’ outcome variable (age of the household head) that is confounded by the same set of unobserved household dynamics (omitted variables) with fertility but lacks any plausible causal association with MHM. Second, to test the methodological sensitivity of our results related to our chosen dependent variable (children ever born, as a measure of individual fertility), and the distributional assumption of the Poisson model. To check the sensitivity of our baseline second-stage Poisson estimation to potential overdispersion, we have replaced the dependent variable with total live births and estimated the second-stage regression with a negative binomial control function. Finally, we checked the boundary conditions of our regression results by stratifying the data across two distinct demographic cohorts of women – rural women and younger women (ages 15-29 years). This subsample analysis helps us to understand the heterogeneity of the causality of MHM on fertility and thereby confirms the robustness of our results.

## Results

### Menstrual hygiene management in Togo

Table 2 indicates that only 57% of women in Togo manage their menstrual hygiene. 32% of rural girls and women manage their menstrual hygiene out of which 83% use reusable materials, and only 17% use single-use materials. For the urban girls and women, the ratio is opposite.

**Table 2.** MHM and WASH facilities in Togo.

Menstrual health management	National (percent)	Urban (percent)	Rural (percent)
Menstrual health practice	57	75	32
A private place to wash and change	93	58	42
Use of menstrual materials-Reusable	62	23	83
Use of menstrual materials- Single-use	36	77	17
Exclusion of participation in activities during menstruation	24	19	28
WASH Facilities			
No water facility	3	1	5
Water available in own dwelling or yard or plot	12	15	10
Water fetching from elsewhere	85	84	85
Place available for hand wash	32	42	25
Hand cleanser available	74	87	65
No toilet facility	42	9	66
Shared toilet with household	53	68	32
Shared toilet with the public	47	14	71

Source: Authors’ calculation from MICS6 data of Togo, 2017.

Information on WASH Data reveals that only 12% of households have water facilities at their dwelling or plot or land in Togo. In urban areas, this percentage is 15 and in rural areas, it is 10%. 85% of households are fetching water from other sources and 65% of rural girls and women reported that hand cleanser materials are available to them. However, the data does not contain any information on whether soap or cleanser is available for cleaning reusable menstrual products or not, as we have realized that 83% of rural women and girls use reusable products during menstruation.

Regarding the toilet facility, 42% of women and girls have reported not having toilet facilities, out of which 66% are residing in rural areas. About 47% of the respondents reported that they are using public toilets, out of which 77% are from rural Togo. These figures portray the real hygiene challenges faced by Togolese girls and women during menstruation. About 28% of rural girls and rural women exclude themselves from social activities during menstruation and generally, 24% of girls and women do so.

#### Socio-economic profile of Togo

Table 3 enumerates the descriptive statistics of the MHM and socioeconomic profile of Togolese girls and women. The data reveals that 63% of Togolese ever married woman between the age group 15-49 years can manage their menstrual hygiene.

**Table 3.** Descriptive statistics.

Variables		Mean	Std. Dev.	Min	Max
*Menstrual hygiene management*		0.63	0.48	0	1
*Fertility*		3.32	2.17	0	14
*Age of the respondents*		32.37	7.84	15	49
*Education level*	Secondary & above	0.35	0.48	0	1
	Primary	0.35	0.48	0	1
	None	0.29	0.46	0	1
*Wealth*	Rich	0.41	0.49	0	1
	Middle/average	0.20	0.40	0	1
	Poor	0.40	0.49	0	1
*Residence type (Rural)*		0.38	0.49	0	1
*Exposure to media/ICT*		0.78	0.41	0	1
*Availability of WASH facilities for maintaining hygiene*	No water, hand washing, and hand cleaning facilities are available	0.02	0.14	0	1
	Water available-own dwelling/yard/plot without a handwashing place and/or hand cleanser	0.10	0.29	0	1
	Water available -elsewhere without a handwashing place and/or hand cleanser	0.23	0.42	0	1
	water available -elsewhere with a handwashing place and/or hand cleanser	0.42	0.49	0	1
	water available - own dwelling/yard/plot with hand washing places and/or hand cleansers	0.24	0.43	0	1
*Use of contraception method*		0.38	0.49	0	1
*Consumption of nicotine (cigarettes/tobacco) or alcohol*		0.75	0.43	0	1
No. of observations: 4989					

*Source: Authors’ calculation based on MICS6 database of Togo, 2017*.

The descriptive statistics (Table 3) reveal that the average age of respondents is 32.4 years. Among ever-married women, the mean fertility rate is 0.40, which is notably low for Togo. The educational profile shows that 29% of respondents are illiterate, 35% have completed primary education, and another 35% have attained secondary education or higher qualifications.

In terms of economic status, the wealth index indicates that 40% of women are classified as poor, 20% belong to the middle class, and 40% fall into the rich category. Regarding menstrual hygiene, 54% of women in the rich class report maintaining proper menstrual hygiene, compared to only 23% among the poor and middle-class groups.

Regarding media and ICT exposure, 78% of women reported access to at least one form of media, such as radio, television, mobile phones, or the internet. However, this exposure does not necessarily translate to social awareness about menstrual hygiene management (MHM), as the data does not provide specific insights into this aspect.

Additionally, 38% of women indicated that they are not using contraception, and 75% reported having consumed alcohol or nicotine (cigarettes or tobacco) at least once.

The water facility data reveals the following insights: 2% of respondents lack access to water, handwashing facilities, and hand cleansers. About 24% reported having water available within their dwelling, yard, or plot, along with handwashing stations and/or hand cleansers. Additionally, 9.5% stated that while water is available in their dwelling, yard, or plot, handwashing facilities and/or hand cleansers are not present. Furthermore, 42% of women indicated that water is accessible elsewhere and is accompanied by handwashing facilities and/or hand cleansers. Lastly, 23% reported that water is available elsewhere but without handwashing facilities or hand cleansers.

## First stage regression results: Factors affecting MHM in Togo

As explained in the previous section, the regression estimations of equation (2) are provided in Table 4. In column 1, we establish the relationship between the WASH facilities available to each individual and their menstrual hygiene management without controlling covariates. We find that the availability of WASH facilities enhances the probability of managing menstrual hygiene by 31 – 53 percentage points (pp). These variables also act as instruments in equation (2).

**Table 4.** Probit regression results (marginal effects).

Variables		(1)		(2)	
		Marginal Effects (95% CI)	p-value	Marginal Effects (95% CI)	p-value
**Instrumental Variables**					
Availability of WASH facility	No water, hand wash, and hand cleaning facility (Ref)	1	-	1	-
	Water available - own dwelling/yard/plot without a handwashing place and/or hand cleanser	**0.31** (0.21, 0.40)	<0.001	**0.20** (0.09, 0.32)	0.001
	Water available -elsewhere without a handwashing place and/or hand cleanser	**0.31**(0.23, 0.40)	<0.001	**0.18** (0.06, 0.29)	0.002
	Water available -elsewhere with a handwashing place and/or hand cleanser	**0.43**(0.34, 0.51)	<0.001	**0.22** (0.11, 0.33)	<0.001
	Water available - own dwelling/yard/plot with hand washing places and/or hand cleansers	**0.53**(0.45, 0.62)	<0.001	**0.20** (0.09, 0.32)	<0.001
**Other Controls**					
Age		-	-	**0.01** (0.003, 0.007)	<0.001
Residence Type	Urban (Ref)	-	-	1	​
	Rural	-	-	**-0.60**(-0.13, -0.02)	0.012
Education level of respondent	Secondary & above (Ref)	-	-	1	-
	Primary	-	-	-0.00 (-0.04, 0.04)	0.991
	No education	-	-	**-0.13** (-0.18, -0.08)	<0.001
Wealth	Rich (Ref)	-	-	1	​
	Middle	-	-	-0.03 (-0.07, 0.01)	0.147
	Poor	-	-	**-0.30** (-0.35, -0.26)	<0.001
Media/ICT exposure		-	-	**-0.07** (-0.12, -0.03)	0.002
Nicotine or alcohol consumption		-	-	0.01 (-0.02, 0.05)	0.481
Household head’s sex	Male (Ref)	-	-	1	-
	Female	-	-	**0.10** (0.06, 0.14)	<0.001
Household head’s age		-	-	**-0.002** (-0.003, -0.000)	0.019
Education level of household head	Secondary & above (Ref)	-	-	1	-
	Primary	-	-	**-0.05**(-0.09,-0.003)	0.035
	No education	-	-	**-0.08**(-0.13,-0.03)	<0.001
*Region and ethnicity dummies*		*No*		*Yes*	
*Log pseudolikelihood*		*-3168.70*	*-*	*-2555.06*	*-*
*Wald chi2*		*211.01*	<0.001	*1276.04*	<0.001
*Pseudo R2*		*0.03*	*-*	*0.22*	*-*
*Observations*		*4,989*	*-*	*4,989*	*-*

*Note.* Constants are not reported. Marginal effects (dy/dx) for factor levels represent the discrete change in probability from the base (reference) level. **Abbreviations:** CI = Confidence Interval; Ref = Reference Category. **Source:** Authors’ calculation.

In column 2, we have added socio-economic and other controls, where we find that the significance and sign of our major variables of concern, WASH facilities, remain unchanged. The coefficients of all categories of water, hand wash, and cleanser facilities indicate that availability of them always increases the probability of menstrual hygiene management of women significantly by 20-22 pp (CI: 0.09, 0.33). As mentioned, we have added the sex of the household head, level of education of the household head, and age of the household head. In the socio-cultural system of Africa, household heads are primarily responsible for adhering to social norms and social exclusions for the smooth functioning of a family.^50^ We have taken the household head’s gender, education level, and age as a proxy for controlling social norms. Our estimation reveals that in the female headed households, the probability of managing menstrual hygiene is 10pp (CI:0.06,0.14) higher than male headed household. As expected, the household head with primary and no education gives relatively less importance (5 pp, CI:-0.09,-0.003, and 8 pp, CI:-0.13,-0.03) lower respectively to MHM than the household head with secondary and above education. Further, an aged household head gives less importance (0.2 pp, CI:-0.003, -0.000) to MHM irrespective of gender.

Further, we have controlled other socioeconomic variables, viz., age of the respondent, educational levels, residential type, wealth, media exposure, region, and ethnicity dummies. In Table 4, we have not reported the results of region and ethnicity dummies for brevity. We find that the probability of menstrual hygiene management increases with women’s age by 1pp (CI: 0.003, 0.007). Non-educated women maintain a relatively low MHM (13 pp, CI:-0.18, -0.08) compared to educated women. Rural women’s management of menstrual hygiene is 60 pp (CI:-0.13, -0.02) than urban women. The probability of managing menstrual hygiene of poor women is 30 pp (CI: -0.35, -0.26) lower than the rich. Media/ICT exposure is having a negative impact (-7pp, CI:-0.12, -0.03) on the probability of MHM. This result is a little surprising but not unexpected. Media exposure does not guarantee social awareness about MHM. It depends on the dissemination of the number of MHM-related awareness advertisements per day in media/internet. It also depends to what extent the girls and women of Togo are listening to or reading MHM-related news given their limited exposure to media and the internet. The negative sign of the coefficient captures this phenomenon to a certain extent.

### Second stage regression results: Effects of MHM on fertility

Table 5 reports the second stage regression where we have hypothesized that MHM has a causal effect on reproductive health outcome, measured in terms of fertility. In column 1, we have reported the poisson estimation results without considering the endogeneity of MHM. The estimation shows MHM enhances the fertility of reproductive-age ever-married women by 4.5% (0.044, CI:0.010, 0.078).

**Table 5.** Second stage regression results.

Variables		MHM (Exogeneous)				MHM (Endogenous)		
		(1) Poisson				(2) CF-2SRI		
Coefficient (95% CI)	p-value		IRR	Coefficient (95% CI)	p-value	lRR		
MHM		**0.044** (0.010, 0.078)	0.011		1.045	**0.052** (0.016, 0.085)	0.004	1.052.
Generalized Residual ( $\hat{m}\left(.\right))$		-	-		-	**1.121** (0.519, 1.722)	0.024	3.016
Age		**0.049** (0.047, 0.051)	< 0.001		1.050	**0.051** (0.049, 0.053)	< 0.001	1.052
Education level of respondent	Secondary & above (Ref)	1	-		-	1	-	-
	Primary	**-0.048** (-0.083, -0.014)	0.005		0.953	**-0.046** (-0.080, -0.012)	0.009	0.955
	No education	**-0.270** (-0.314, -0.225)	< 0.001		0.763	**-0.337** (-0.394, -0.281)	< 0.001	0.714
Residence Type	Urban (Ref)	1	-		-	1	-	-
	Rural	-0.008 (-0.064, 0.049)	0.794		0.992	-0.030 (-0.089, 0.028)	0.313	0.970
*Wealth*	Rich (Ref)	1	-		-	1	-	-
	Middle	**-0.091** (-0.131, -0.051)	< 0.001		0.913	**-0.088** (-0.128, -0.048)	< 0.001	0.916
	Poor	**-0.236** (-0.289, -0.183)	< 0.001		0.790	**-0.348** (-0.428, -0.269)	< 0.001	0.706
Media/ICT exposure		-0.018 (-0.052, 0.016)	0.294		0.982	**-0.030** (-0.065, 0.005)	0.092	0.971
Nicotine or alcohol consumption		**-0.029** (-0.062, 0.004)	0.085		0.971	**-0.028** (-0.061, 0.005)	0.096	0.972
Contraception use		**0.130** (0.103, 0.157)	< 0.001		1.139	**0.129** (0.102, 0.156)	< 0.001	1.138
Household head’s sex	Male (Ref)	1	-		-	1	-	-
	Female	**0.235** (0.196, 0.274)	< 0.001		1.265	**0.267** (0.223, 0.310)	< 0.001	1.305
Household head’s age		**-0.004** (-0.006, -0.003)	< 0.001		0.996	**-0.005** (-0.006, -0.004)	< 0.001	0.995
Education level of household head	Secondary & above (Ref)	1	-		-	1	-	-
	Primary	**-0.046** (-0.083, -0.009)	0.015		0.955	**-0.057** (-0.094, -0.019)	0.003	0.945
	No education	**-0.086** (-0.127, -0.044)	< 0.001		0.918	**-0.108** (-0.151, -0.065)	< 0.001	0.898
*Region and ethnicity dummies*		*Yes*				*Yes*		
*Pseudo R-squared*		*0.1639*	-	-		*0.1643*	-	-
*Wald chi2*		*5027.43*	*<0.001*	-		*5082.28*	*<0.001*	-
*AIC*		*18038.06*	-	-		*18030.64*	-	-
***IV Diagnostic Statistics***								
Sargan-Hansen J statistics ( $\chi^{2}$ )		-	-	-		*3.353*	*0.3404*	-
*Kleibergen-Paap rk LM statistic*		-	-	-		*21.692*	*0.0002*	-
*Weak identification Test: Kleibergen-Paap Wald F statistic*		-	-	-		*16.010*	-	-
Observations		*4,989*				*4,989*		

*Note.* Constants are not reported. **Dependent variable**: Children ever born. **Abbreviations:** CI = Confidence Interval; Ref = Reference Category; CF-2SRI = Two-Stage Residual Inclusion (2SRI) control function approach; IRR: Incidence Rate Ratio. Coefficients of variables of Poisson regression cannot be interpreted directly. The IRR is calculated as: IRR = $e^{\beta }$ . Hence, the percentage change effect of an explanatory variable is calculated as ( $e^{\beta }$ -1)*100%. The CF model uses WASH facilities as instrumental variables. **Source:** Authors’ calculation.

Column 2 represents the second stage results of two-stage residual inclusion (2SRI) control function estimation. The instruments that we have considered here are the availability of water for other purposes (other than drinking and household use), location of water source, availability of hand-washing place, and availability of any hand cleanser (explained in first stage regression results, Table 4). The significance of the generalized residual, $\hat{m}$ , in CF-2SRI estimate ensures that the MHM is endogeneous. After addressing the endogeneity of MHM, we see that the coefficient of MHM remains positive and significant, which indicates better menstrual hygiene management enhances the reproductive health (fertility) of women by 5.2% (0.052, CI: 0.016, 0.085). We have controlled the age of the respondent, residence type, education levels of the respondent, wealth, age, sex, education levels of household head, regions, ethnicity, contraception use, consumption of nicotine and alcohol, and media/ICT exposure. After controlling the endogeneity of MHM, we find that age, education, wealth, consumption of nicotine and alcohol, female-headed household and their education have significant impacts on women’s fertility. Non-educated women have 28.6% (-0.337, CI: -0.394, -0.281) lower fertility than educated women. Also, the middle-income and poor-income group of women have lower fertility by 8.4% (-0.088, CI: -0.128, -0.048), and 29.5% (-0.348, CI:-0.428, -0.269) respectively than the rich women. Females who do not use any contraception method have higher fertility by 13.8% (0.129, CI: 0.102, 0.156). Women of reproductive age have a higher fertility rate by 30.5% (0.267, CI: 0.223, 0.310) in female-headed households, whereas their fertility is low by 10.2% (-0.108, CI:-0.151, -0.065)) when the household head is illiterate, and by 5.5% when the household head’s education is at primary level compare to qualified (secondary & above) heads. Exposure to media reduces the individual level fertility by 2.9% (-0.030, CI: -0.065, 0.005), and also consumption of nicotine and alcohol has negative impact on fertility by 2.8% (-0.028 CI: -0.061, 0.005).

### Instrument validity tests

In order to test the relevance, validity and strength of the instruments, we have performed following post-estimation tests as a part of standard regression diagnostics on the full sample: (1). Kleibergen-Paap rk LM test statistic is 21.69 (p = 0.0002), which strongly reject the null of underidentification and validates that the instruments are strongly correlated with the endogeneous regressor, MHM; (2) the Kleibergen-Paap rk Wald F statistic is 16.01, which significantly exceed the 10% bias threshold level (10.27),^51^ ruling out the null of weak instruments; and finally, (3) the Sargan-Hansen J statistic ( $\chi^{2}$ ) is 3.353(p = 0.3404) indicates that the null of the validity of our instruments is accepted.

### Placebo test

To ensure the validity of the identification strategy and to confirm the exclusion restriction of the instrumental variables, we conduct the Placebo tests.^49^ The result is shown in Supplemental Table S1. The CF-OLS results validate our exclusion restriction of the identification strategy. The coefficient of MHM is –2.28 (CI: -8.64,4.09, p = 0.483), and the insignificance of the generalized residual, $\hat{m}$ (0.84, CI: -2.88,4.56, p = 0.656) rules out the endogeneity of MHM. These imply no unobserved confounding bias links MHM to the age of the household head.

Further, in IV-2SLS model, the coefficient of MHM is -7.48 (CI: -17.22, 2.26, p = 0.132), turns out to be insignificant, failing to reject the null, confirming the exclusion restriction. The Kleibergen-Paap rk LM statistic (22.08, p = 0.0002) confirms that the instruments (WASH facilities) are strongly correlated with MHM. Further, the Hansen J test (Chi-sq = 5.11, p = 0.164) validates that the instruments are strictly exogenous.

### Sensitivity analysis

As mentioned in the statistical methods section, to perform the sensitivity analysis of our baseline estimates, we have replaced the dependent variable with total live births as a proxy of individual-level fertility. We have further used negative binomial (NB) regression to account for potential overdispersion in count data. The NB model parameterises the conditional variance as a quadratic function of mean (μ) , $var\left(y|x\right)=\mu +\alpha \mu^{2}$ captures the degree of overdispersion driven by unobserved heterogeneity.^52^ Our estimation indicates that α (overdispersion parameter) converges to zero (1.83e-09), confirming that conditional variance does not exceed the conditional mean, that is, var(y|x)=E(y|x)=μ , which satisfies the equidispersion assumption of Poisson regression. This validates the Poisson specification of the baseline model. Further, the coefficient of MHM (0.034,CI: -0.001,0.068) and the significance of generalised residual (-0.499, CI: -0.863, -0.135) validates the robustness of our baseline results (see Supplemental Table S2).

### Heterogeneity analysis

We have further performed the subsample analyses to check the robustness of our results (see Supplemental Table S3). The subsample we have selected based on our regression results is shown in Table 5. The following subsamples we have considered for our robustness check analysis: column 1 reports the second-stage CF-2SRI estimates and results of the subsample of Younger Women (Age Group between 15 and 29). Column 2 shows the second-stage CF-2SRI estimates of the subsample of the respondents who reside in the rural areas of Togo. The dependent variable is the children ever born, and the instruments are WASH facilities. In both specifications, we have controlled the socioeconomic factors as considered in Table 5. We haven’t reported those estimates for brevity. In both subsample analyses, the significance of the generalized residual ( $\hat{m})$ ensures the endogeneity of MHM. Furthermore, the Sargen-Hansen J statistics reflect the validity of the instruments at the subsample level. In both subsamples, we find that MHM has a significant positive impact on women’s fertility after controlling for endogeneity. These ensure the robustness of our baseline regression estimates.

## Discussion

Managing menstrual health safely and appropriately can be achieved by providing health education, identification, and treatment of menstrual disorders to women and girls.^53,54^ However, the pervasive stigma of menstruation and entrenched social norms limit adequate support for menstruating girls and women,^44^ making MHM a multisectoral policy challenge affecting sexual and reproductive health, education, water, sanitation, and hygiene (WASH), and so on.^6,53,55^ Improving MHM is complicated further due to access to menstrual hygiene commodities, and effective and successful management of menstrual hygiene requires women and girls to have access to sufficient quality and quantity of menstrual health products to effectively manage and maintain their menstrual cycle.^5,6,56^ The issue is even more relevant in developing countries, particularly in the Global South. Poverty and the lack of suitable places offering the necessary privacy for women to change and/or wash, such as for schoolgirls, exacerbate the problems associated with menstrual hygiene management at home.^57^ In most cases, poor hygiene practices and housekeeping issues are caused by insufficient WASH facilities.^32,58^ In a similar vein, our findings indicate that access to water, hand-washing facilities, and hand-sanitizing supplies are crucial elements in the management of menstrual hygiene. 12% of respondents of Togo report that water is available at their dwelling/yard/plot, and 23.7% have hand cleansers and hand-washing places. Our regression results have indicated that these availabilities increase the chances of MHM by 31 – 53 percentage points.

Further cross-national studies indicate that socioeconomic inequality and sociodemographic factors affect MHM significantly.^49,58–62^ Our study reflects the same in the case of Togo. Our findings suggest that a woman’s age, education level, and wealth status significantly influence how she manages menstrual hygiene at the household level. Additionally, broader socioeconomic factors such as the sex, age, and education level of the household head, as well as the type of residence, also play a crucial role. We find that poor, illiterate, primary-educated individuals and rural women manage their menstrual hygiene very poorly compared to rich and middle-class, educated, and urban women.

The main contribution of our study is to examine the causal impact of MHM on reproductive health outcomes, measured in terms of fertility of Togolese women. Existing studies have not explored this causal channel but have evidenced that poor menstrual hygiene leads to reproductive morbidity. The cross-national studies also establish the channel through which MHM affects reproductive health inversely,^13–19^ but none of them captures the causal linkage. Our study finds a strong causal effect of MHM on fertility. We have accounted the endogeneity of MHM successfully by employing 2SRI control function approach through the channels of household infrastructure like WASH facilities. The baseline estimate reveals that effective MHM increases fertility by 5.2%. This can be explained through the possible channels of better MHM reduces RTIs/STIs, which in turn reduces infection-driven infertility and improves fertility, the channel which has been explained in the existing literature.^20–23^ UNFPA (2021)^63^ evidenced a bi-directional causality between menstrual health and sexual and reproductive health, and the pathways explained from biological and social points of view support our findings.

Our study also controlled the socioeconomic constraints, household dynamics, and female autonomy while explaining this causal pathway. After controlling the endogeneity of MHM, we find that age, education, wealth, consumption of nicotine and alcohol, female-headed household and their education have significant impacts on women’s fertility, consistent with the studies in the context of sub-Saharan Africa.^64,65^

We find that uneducated women, and those belonging to the poor wealth quintile, exhibit lower childbirth/fertility compared to their educated and wealthier counterparts. While the classical demographic transition theory^66^ indicates that higher income and education reduce fertility through an increase in the opportunity costs and shifting preferences of women, recent cross-sectional studies evidence a heterogeneous impact of these socioeconomic factors on fertility. A U-shaped relationship exhibited by Ketkar (1978)^67^ wherein poorly educated women may exhibit low fertility. Further, the inherent channels of this low fertility/infertility in sub-Saharan Africa have been explained in many studies, such as lack of resource setting,^68^ Assisted Reproductive Technologies(ART) infrastructure, health education,^69^ access to healthcare^70^ and stringent social norms in low wealth and uneducated cohorts.^71,72^ In a resource constraint setting, poverty can suppress the realised fertility through the channels of higher prevalence of subfecundity, malnutrition, and other socioeconomic constraints.

Our regression results also indicate that ICT/media exposure has a negative impact on MHM. The possible reasons include a lack of awareness, limited broadcasting/media coverage, or relatively small numbers of awareness programs related to MHM in the media. This entails a requirement of advocacy and awareness programs at a community level to disseminate knowledge regarding the benefits of MHM and its positive impact on reproductive health and destigmatizing menstruation. Further, our second-stage regression results that media exposure reduces the fertility of women. This supports the well-documented ideational effect of mass media in family planning. Access to media increases women’s health literacy, exposes them to the family planning methods, and standardises smaller family norms; therefore, effectively expediting the reproductive behavior of women independent of formal schooling.^73^

Thus, this study helps to identify and talk about at least three overlapping elements that are required to be accessed to achieve good menstrual hygiene and reproductive health in Togo: (a) accessibility of sexual and reproductive health-related knowledge and information to comprehend the natural menstrual cycle and to overcome stigma and misleading information; (b) accessibility to high-quality, affordable menstrual products; and (c) access to women-friendly facilities, which is essential for menstruating women to have a safe and private place to change, and meet their hygiene needs. These factors are influenced by an *enabling environment*, guided by public policies, laws, processes, and business environs to influence the availability and pricing of products, discriminatory practices in social prejudice, or standards of public health design.

## Limitations

The study has a few limitations: the survey data captures the self-reported menstrual hygiene behaviour of individual woman during their last menstrual period. It captures the environmental hygiene, but cannot capture the actual quantity of hygiene, for example, the frequency of changing materials, etc. Hence, a crude, adequate MHM indicator is constructed, which is not free from potential social desirability bias in menstrual hygiene behaviour. Further, the survey data lack clinically relevant biomarkers of the frequency of RTIs/STIs during the period, limiting econometric analyses of the channels linking low fertility to RTIs/STIs due to poor MHM. Finally, the information related to WASH does not report explicit use of water or washing facilities during menstruation. Hence, it remains challenging to isolate the WASH infrastructure available for maintaining menstrual hygiene from the household-level WASH infrastructure. Availability of the above-mentioned information could help to construct perfect strong instruments to get more precise estimates in establishinga causal linkage between MHM and fertility.

## Conclusion

Menstrual hygiene management remains a neglected issue in West African countries, and so in Togo. There exists very thin literature that discusses the menstrual hygiene practices in Togo and its impact on reproductive health, measured in terms of fertility. To the best of our knowledge, our study is the first attempt to investigate the causal impact of menstrual hygiene management on individual-level fertility, measured in terms of child ever born by using a large sample dataset.

Our estimates reveal that WASH infrastructure is necessary to improve MHM. The magnitude increase is between 31-53 percentage points. The estimations also establish a strong causal relationship between MHM and female fertility, estimating a 5.2% increase in fertility due to effective MHM, even though disparity is observed between rural and urban women and between young (15-29) and older women (30-49) cohorts. We also find that age, education, wealth, consumption of nicotine and alcohol, female-headed household and their education have significant impacts on women’s fertility in Togo.

## Supplemental material

Supplemental material - Menstrual hygiene management and fertility in Togo: Exploring the causal pathways Supplemental material for Menstrual hygiene management and fertility in Togo: Exploring the causal pathways by Anusree Paul, Salmata Ouedraogo and Anastasie Amboulé Abath in Women’s Health.

Supplemental material - Menstrual hygiene management and fertility in Togo: Exploring the causal pathways Supplemental material for Menstrual hygiene management and fertility in Togo: Exploring the causal pathways by Anusree Paul, Salmata Ouedraogo and Anastasie Amboulé Abath in Women’s Health.

## Data Availability

The data underlying the results presented in the study are available from https://mics.unicef.org/surveys.
