# Supplementary material for: Menstrual hygiene management and fertility in Togo: Exploring the causal pathways
Source: Womens Health (Lond). 2026 Jun 4;22:17455057261458614. doi: 10.1177/17455057261458614 (PMC13237468; doi:10.1177/17455057261458614)
Supplement: Supplemental material - Menstrual hygiene management and fertility in Togo: Exploring the causal pathways [file sj-pdf-2-whe-10.1177_17455057261458614.pdf]

Table S1: Placebo Test Results

	Placebo Test			
	(1) CF-OLS		(2) IV-2SLS	
**Variables**	**Coefficient (95% CI)**	**p -value**	**Coefficient (95% CI)**	**p -value**
MHM	-2.28 (-8.64,4.09)	0.483	-7.48 (-17.22, 2.26)	0.132
Generalized Residual $(\widehat{m}(.))$	0.84 (-2.88,4.56)	0.656	-	-
Other covariates	*Yes*		*Yes*	
R-squared	*0.252*	*-*	*0.202*	*-*
F stat	*70.08*	*<0.001*	*67.6*	*<0.001*
Sargan-Hansen J statistics (χ^2)	-	-	*5.113*	*0.164*
Kleibergen-Paap rk LM Statistics	-	-	*22.084*	*< 0.001*
Weak identification Test: Kleibergen-Paap Wald F statistic	-	-	*16.130*	-
Observations	*4989*			

. ***Note:*** *Constants are not reported.* ***Dependent variable****: Age of household head.* ***Abbreviations:*** *CI = Confidence Interval; Ref = Reference Category; OLS = Ordinary Least Squares; CF = Control Function estimates (2nd stage).The CF model uses toilet facilities, and handwash facilities as instrumental variables.*
Source: *Authors' calculation*

Table S2: Sensitivity Analysis

**Variables**	**Coefficient (95% CI)**	**p-value**
MHM	0.034 (-0.001,0.068)	0.053
Generalized Residual $(\widehat{m}(.))$	-0.499 (-0.862, -0.135)	0.007
Region and ethnicity dummies	*Yes*	
Other controls	*Yes*	
Pseudo R-squared	*0.1495*	-
Wald chi2	*4899.14*	*<0.001*
alpha	*1.83e-09*	-
Observations	*4904*	

Table S3: Robustness Check Results (CF-2SRI) – Sub-Samples

**Sub-Samples Groups**	**(I). Respondents: Younger Women (Age Gr. 15 to 29)**			**(II). Rural Women**		
**Variables**	**Coefficient (95% CI)**	**p -value**	**IRR**	**Coefficient (95% CI)**	**p -value**	**IRR**
MHM	**0.131** (0.056, 0.206)	0.001	1.140	**0.050** (-0.003, 0.103)	0.066	1.051
Generalized Residual $(\widehat{m}(.))$	**-0.657** (-1.057, -0.258)	0.001	0.518	**-0.193** (-0.390, 0.005)	0.056	0.825
Other covariates	*Yes*			*Yes*		
Pseudo R-squared	*0.3447*	-	-	*0.3088*		-
Wald chi2	*4258.28*	*<0.001*	-	*40.85*	*<0.001*	-
Sargan-Hansen J statistics (χ^2)	*3.979*	*0.2638*	-	*5.627*	*0.1312*	-
Kleibergen-Paap rk LM Statistics	*27.542*	*<0.001*	-	*22.494*	*0.0002*	-
Weak identification Test: Kleibergen-Paap Wald F statistic	*16.983*	-	-	*16.937*	-	-

Observations	*1977*	*1879*

Note: *Constants are not reported.* ***Dependent variable****: Children ever born.* ***Abbreviations:*** *CI = Confidence Interval; CF-2SRI = Two-Stage Residual Inclusion (2SRI) control function approach; IRR: Incidence Rate Ratio. Coefficients of variables of Poisson regression cannot be interpreted directly. The IRR is calculated as: IRR =* e^{β}*. Hence, the percentage change effect of an explanatory variable is calculated as (*e^{β}*-1)*100%. The CF model uses WASH facilities as instrumental variables.*

Source: *Authors' calculation*
